# Deep learning for Angle classification based on intraoral photographs: an interpretability perspective

**DOI:** 10.1186/s12903-025-07550-6

**Published:** 2026-01-04

**Authors:** Petra Julia Koch, José Eduardo Cejudo Grano de Oro, Martha Büttner, Lubaina Tayeb Arsiwala-Scheppach, Julia De Geer, Henrik Meyer-Lueckel, Falk Schwendicke

**Affiliations:** 1 Department of Prosthodontics, Geriatric Dentistry and Craniomandibular Disorders, CharitéCenter for Oral Health Sciences CC3, Charité – Universitätsmedizin Berlin, corporate member of Freie Universität Berlin and Humboldt-Universität Zu Berlin, Berlin, Germany; 2 Orthodontic Practice, Berlin, Germany; 3 Department of Oral Diagnostics, Digital Health and Health Services Research, CharitéCenter for Oral Health Sciences CC3, Charité – Universitätsmedizin Berlin, corporate member of Freie Universität Berlin and Humboldt-Universität Zu Berlin, Berlin, Germany; 4 Department of Orthodontics and Dentofacial Orthopedics, CharitéCenter for Oral Health Sciences CC3, Charité – Universitätsmedizin Berlin, corporate member of Freie Universität Berlin and Humboldt-Universität Zu Berlin, Berlin, Germany; 5 Department of Restorative Preventive and Pediatric Dentistry, zmk Bern, University of Bern, Bern, Switzerland; 6 Clinic for Conservative Dentistry and Periodontology, University Hospital of the Ludwig-Maximilians-University Munich, Munich, Germany

**Keywords:** Angle classification, Artificial intelligence, Deep learning, Digital orthodontics, Digital photography, Orthodontic malocclusions

## Abstract

**Background and objective:**

Intraoral photographs are routinely taken in orthodontic practice and provide valuable visual information for diagnostic purposes. To support the training of dental graduate students and prospective orthodontists in diagnosing sagittal malocclusions, this study aimed to develop an artificial intelligence (AI) model to classify sagittal dental malocclusions from digital intraoral photographs using the widely accepted Angle classification system, and to evaluate the explainability of the model’s decisions using three explainable AI (XAI) methods.

**Materials and methods:**

A total of 5266 clinical RGB images showing dental occlusion from the lateral view of first-time orthodontic patients were retrieved from the clinic’s image database and classified by orthodontic experts into Angle Class I (2322 images; 44%), Class II (1880 images; 36%), and Class III (1064 images; 20%).The dataset was then divided into a training set of 4280 images. The validation set contained 474 images, and the test set comprised 512 images of a deep-learning classification model (VGG-11). The employed deep-learning classification model (VGG-11) was then trained and evaluated. Three XAI methods (Layerwise Relevance Propagation, PatternNet, and PatternAttribution) were used to generate heatmaps highlighting relevant areas for classification.

**Results:**

The deep learning model correctly classified 75% of the test set images, achieving high predictive performance across all three Angle classes, with the area-under-the-curve being 0.91, 0.90, and 0.91 for Angle classes I, II, and III, respectively, indicating substantial discriminative ability. The most frequent misclassifications were Angle Class I being misclassified as Angle Class III, and Angle Classes II and III as Angle Class I. XAI highlighted the area surrounding the first molars as decisive for classification, although the three different XAI methods utilized different areas.

**Conclusion:**

Deep learning proved effective for classifying dental malocclusion into Angle classes I, II and III using intraoral photographs. XAI revealed that the classification was based on clinically relevant features. Different XAI methods reflected on different features; combining more XAI methods may allow comprehensive assessment of a model’s classification logic and may accelerate the transfer into clinical application.

## Introduction

Computer vision based on deep learning has become the dominant paradigm for the automated analysis of visual data, particularly with Convolutional Neural Networks (CNNs) achieving state-of-the-art performance in image classification and segmentation. In dentistry, computer vision has been applied to automate the detection of pathological lesions on radiographs [2], but also a range of other applications [24, 33]. In orthodontics, the focus has been on automated cephalometric landmark detection to standardize the difficult and labor-intensive landmarking process [12, 14], Felix [18], F. [19, 21, 23, 25, 26, 32, 36, 38] and assist decisions-making for orthodontic treatments [12], Felix [18, 23, 35], orthognathic surgery outcomes [9, 37], growth determination and development periods [12, 17], Felix [18, 20, 23, 27, 30, 39]. Recently, efforts have extended to creating automated orthodontic treatment plans and simulations [25, 31, 34].

Despite their success, CNNs pose challenges due to their complexity and "black box" nature, making it difficult to interpret their inner workings [6]. To address this, Explainable AI (XAI) methods are used to produce heatmaps that highlight relevant areas for classification, helping developers and users understand the model's logic. These insights could then be utilized to further improve the model through an iterative feedback loop. Generally, XAI techniques decompose input images into signals (relevant pixels) and distractors (irrelevant pixels), with relevance scores indicating the importance of each pixel for the imposed classification outcome [10].

To implement these methods and further expand the potential of AI in supporting dental graduate students and prospective orthodontists in diagnosis and treatment planning [13], commonly available digital data from orthodontic practices – such as digitally captured intraoral photographs, which are routinely used for documenting and tracking the patient’s treatment progress [15] – can be utilized. This data modality—namely intraoral photographs—has so far been used in only two studies involving the application of AI methods: one for predicting unerupted premolars and canines [3], and another for classifying orthodontic records for archiving [22]. However, it also holds potential for more complex tasks, such as classifying a patient’s sagittal occlusion based on the widely recognized Angle classification system, and ultimately to determine the need for orthodontic treatment [1].

**Fig. 1:**
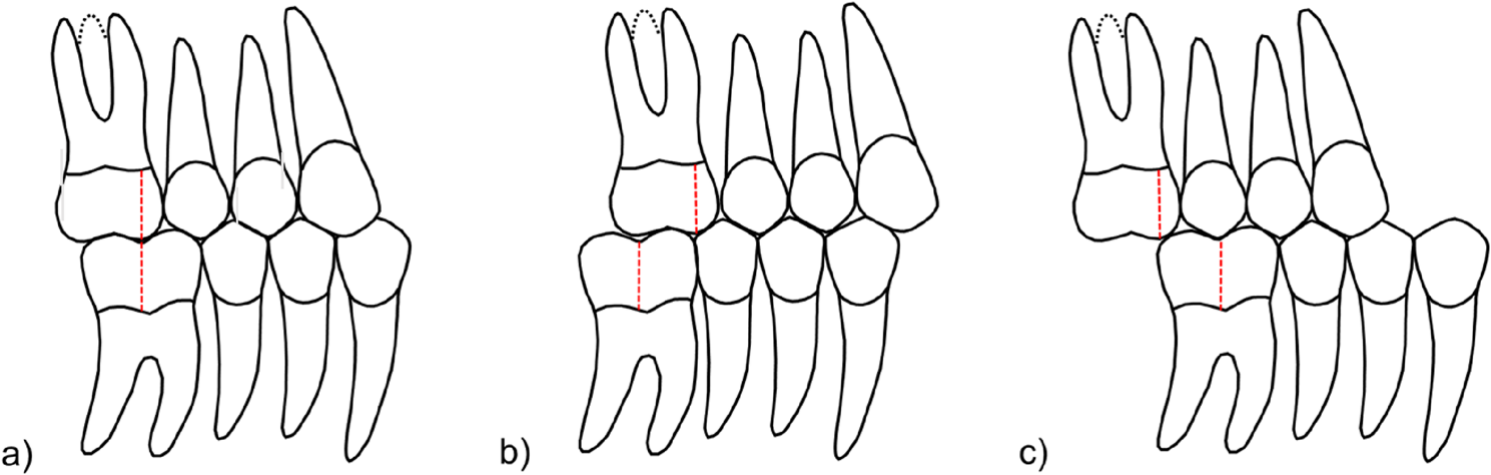
Schematic representation of the Angle classification: **a** Angle class I, **b** Angle class II and **c** Angle class III

The Angle classification is an essential component of the diagnostic routine, alongside recording the patient’s medical history and performing a clinical examination, contributing to a comprehensive orthodontic diagnosis. Established by Edward H. Angle, it qualitatively describes the patient’s sagittal occlusal relationship between the upper and lower first molars, categorizing them into different types of malocclusions [4, 29]. Angle Class I represents a neutral occlusion, where the mesiobuccal cusp of the upper first molar occludes in the groove between the buccal cusps of the lower first molar, with the remaining the teeth aligned accordingly [1]. Angle class II indicates a more distal position of the groove between the cusps of the lower first molar in relation to the mesiobuccal cusp of the upper first molar, whereas Angle class III represents a more mesial position of the groove (Fig. 1). Alongside this, a quantitative assessment is typically performed using measurements of half a cusp, greater than half a cusp, and a full cusp. The qualitative and quantitative differentiation between Angle classes is clinically significant, particularly in cases of malocclusion. When the deviation exceeds the width of a single premolar, such as in a half-cusp deviation where the cusps meet directly, the mandible must be repositioned to establish a functional occlusion. This may lead to unphysiological tooth wear, functional limitations, and discomfort or pain in the temporomandibular joint.

The clinical significance of this study lies in the exploration of the potential of AI to enhance the efficiency and accuracy of Angle class classification. By automating this process, AI can reduce variability across different clinicians and standardize assessments, thereby making the diagnostic process more reliable. Moreover, integrating the use of AI with digital intraoral photographs allows for precise and consistent documentation of occlusion throughout orthodontic treatment. This supports longitudinal monitoring and enables timely interventions at critical stages, which can facilitate adjustments to treatment modalities. Additionally, it supports remote consultations and enhances clinical infrastructure by enabling more systematic archiving. AI can also support clinical education by providing real-time, objective feedback to students and young practitioners, thereby improving learning and decision-making. Moreover, AI’s ability to analyze malocclusions could contribute to more individualized treatment plans.

In light of these benefits, the aim of this study was to develop a deep learning model for classifying sagittal dental malocclusions according to the widely used Angle classification, based on intraoral lateral photographs. Additionally, we evaluated and compared three explainable artificial intelligence (XAI) algorithms to assess their interpretability and utility, addressing the growing need for transparency and accountability in AI applications within dentistry.

## Materials and methods

### Study design

**Fig. 2:**
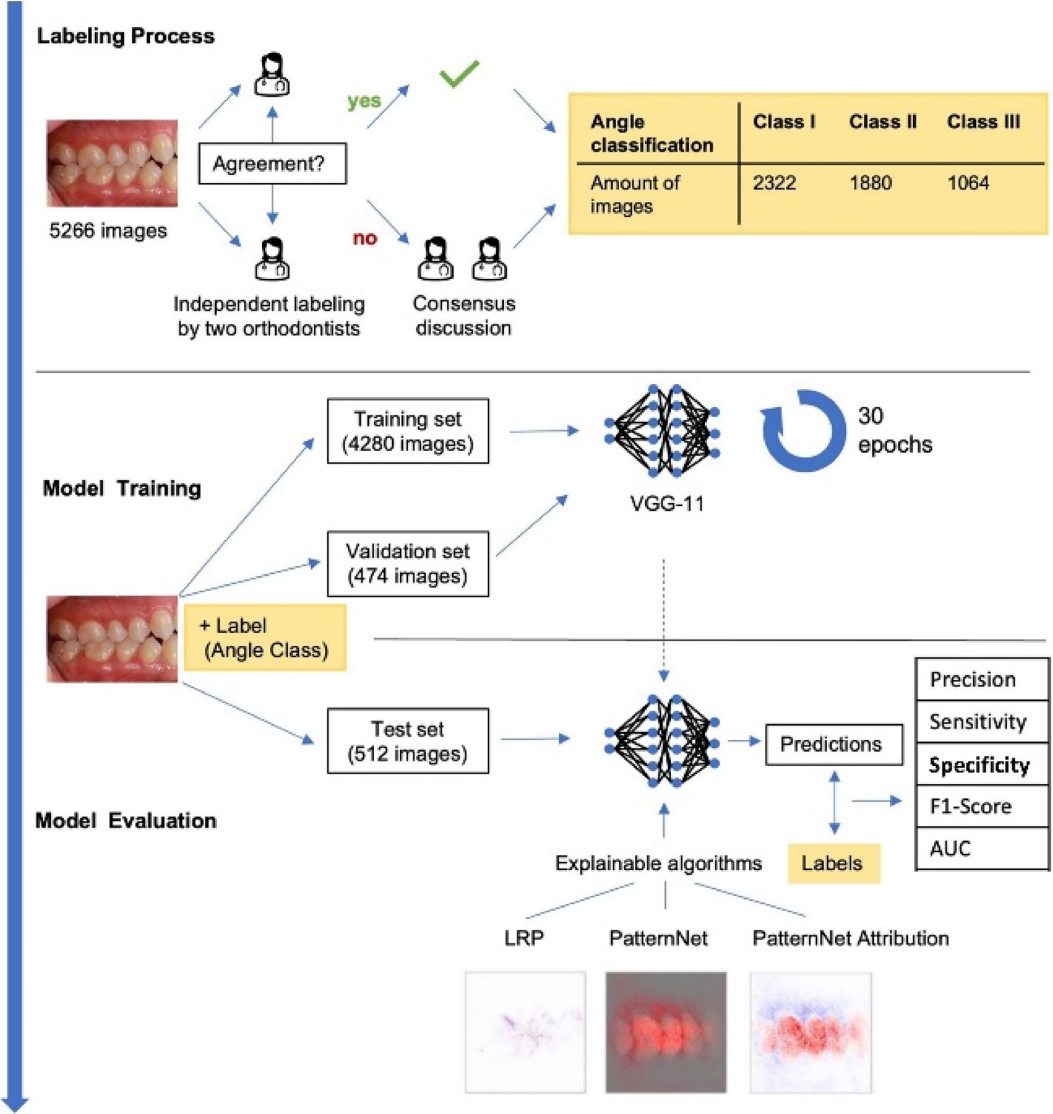
Study workflow. First the labeling process was performed by two orthodontists as indicated. The data set was divided in training and validation set, used to train a VGG-11 model for 30 epochs and a separate test set. The latter was used in model evaluation: the trained model was evaluated in two ways: (1) Performance metrics were computed and (2) explainable algorithms were applied

In this single-centered and retrospective study, the diagnosis of Angle classes in clinical images was approached as an image classification problem and three XAI methods were applied to visualize the model’s decision making. Figure 2 illustrates the entire study workflow: The dataset was divided into training, validation, and test sets, which were assembled and validated by two certified orthodontists who annotated the images with labels representing the different Angle classes. The model was trained and validated and its performance on the test set was evaluated. Subsequently, various XAI methods were employed to make the model’s decisions explainable.

### Data and sampling

Prior to this study, data collection was ethically approved (EA4/080/18) by the local ethics commission. The dataset consisted of 5266 clinical RGB images retrieved from OnyxCeph^3^™ software (Image Instruments, Chemnitz, Germany) at the Department of Orthodontics and Dentofacial Orthopedics. There were 42.1% male and 57.9% female patients; the mean (SD) age was 18.9 (10.6) years. However, gender and age were not considered relevant to the study, as only images showing permanent dentition were used. Mixed dentitions were excluded to avoid confusion with physiological distal relationships of first molars during this period, as the jaw's leeway space, resulting from the discrepancy in mesio-distal width between the deciduous and permanent premolars and canines, could introduce potential confounding factors. Attendings and postgraduate students, instructed in taking intraoral photographs for clinical purposes, captured all images using the same reflex camera (Canon 80D, Ota, Japan) with a Canon macro lens and identical settings (focal length 1/200, aperture 22). The images showed the sagittal dental occlusion from the lateral view (right and left; the left mirrored to the right for each patient) and were reviewed and classified independently by two certified orthodontic experts, who considered only the position and sagittal relationship of the upper and lower first molars, as originally stated by Angle. If no initial agreement was reached on the respective Angle class, a consensus discussion was conducted, and a final diagnosis was determined jointly. Since the model was trained to solve an image classification problem based solely on the presented information in the photograph, an anticipated reconstruction of the sagittal occlusion, as originally proposed by Angle for a correct classification of the presented malocclusion, was not considered. This approach was avoided to prevent making the task overly complex for the model, and the two orthodontic experts were instructed accordingly.

The dataset excluded images with missing teeth, large restorations, and brackets (Table 1). It included 2322 images categorized as Angle Class I (44%), 1880 as Angle Class II (36%), and 1064 as Angle Class III (20%). In cases where image angles were suboptimal, classifications were verified by two orthodontic experts to establish a consensus-based ground truth. The dataset was then divided into a training set of 4280 images, a validation set of 474 images, and a test set of 512 images, maintaining the same distribution of Angle classes across all sets. The dataset was randomly split into training, validation, and test sets while maintaining proportional representation of each Angle class to ensure class balance. This dataset selection aligns with standard practices in dental machine learning research, where most studies partition data into training and testing subsets In a recent scoping review, Arsiwala-Scheppach et al. [5] reported a median training set size of 450 for machine learning applications in dentistry. Although no formal sample size calculation was performed, all available images that met the inclusion criteria and were accessible from the department’s image platform were included, ensuring comprehensive data utilization.

**Table 1:** Inclusion and exclusion criteria

	**Inclusion criteria**	**Exclusion criteria**
Clinical aspects	- Permanent dentition - Dental status prior to orthodontic treatment	- Deciduous teeth - Asymmetrical extractions in the upper and lower jaws - Previous or present orthodontic treatment - Missing permanent upper lateral incisors - Syndromes and cleft lip/palate - Severe tooth wear - Extensive prosthodontic rehabilitation - Open mouth (not open bite)
Technical aspects	- Visible first upper and lower molars - (Near) Rectangular angle of the lateral sagittal occlusion	- No display of molars - Severely acute photo angulation - Blurry photo - Excessive saliva

### Data preparation, model and training

**Fig. 3:**
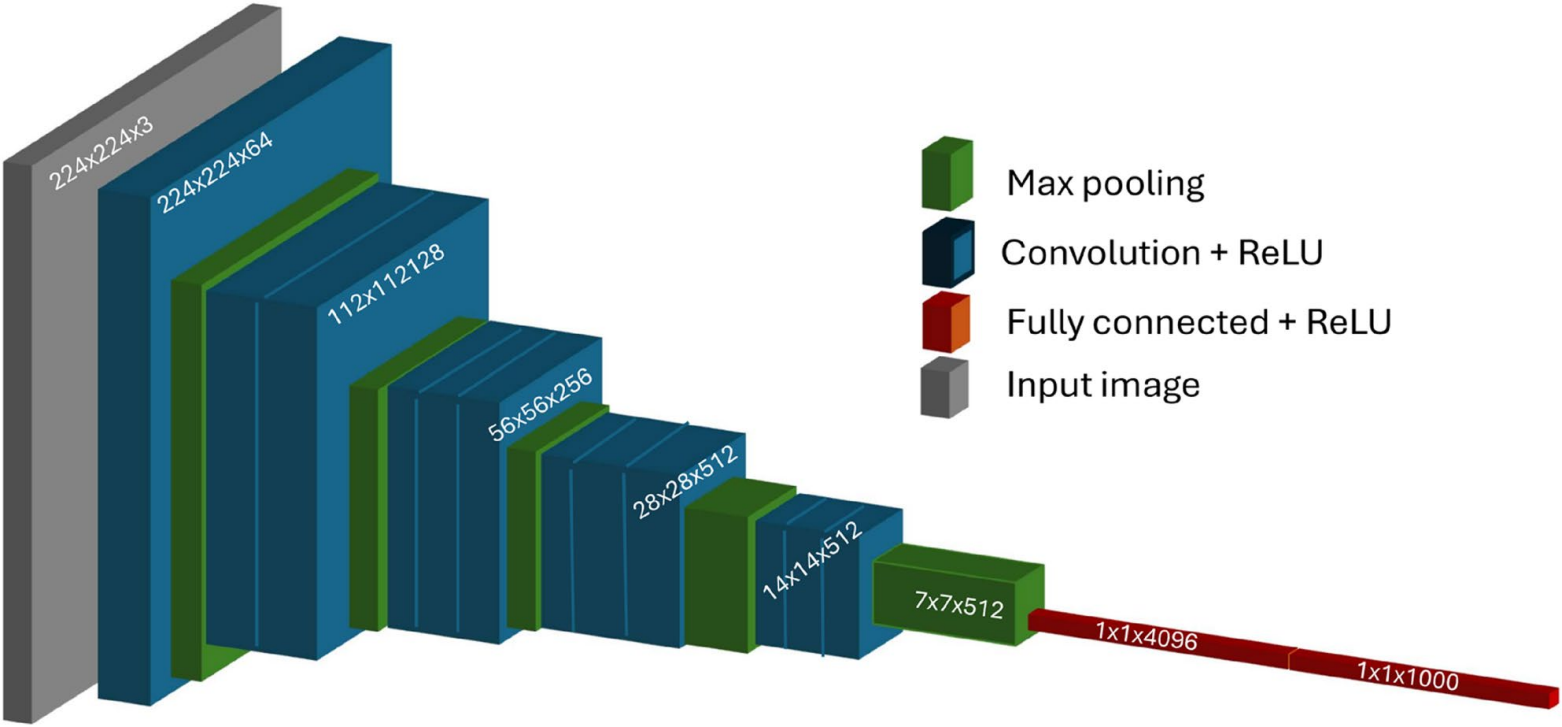
VGG-11 architecture. VGG-11 is a convolutional neural network composed of 11 layers with learnable weights, including 8 convolutional layers followed by 3 fully connected layers. It uses small 3 × 3 convolution filters stacked sequentially with max-pooling layers to gradually reduce spatial dimensions while capturing hierarchical image features

A VGG-11 deep learning architecture pretrained on the ImageNet dataset was utilized [11]. A classification head with output neurons matching the number of categories was added, followed by a SoftMax activation function. The input to the model was an RGB image, and a probability distribution over the categories was output, serving as confidence scores. To address class imbalance, a weighted cross-entropy loss function with weights inversely proportional to each category's frequency was used, penalizing misclassifications of underrepresented categories. The reason behind choosing VGG-11 (Fig. 3) over more complex architectures are its simple, purely sequential architecture which makes more interpretable saliency maps. Its smaller size speeds up per-sample explanations and layer-wise visualizations, works better with smaller datasets by avoiding overfitting, and making the results comparable with other works on XAI, making it a reproducible choice when understanding of model decisions are more important than state-of-the-art accuracy.

Images were resized to 256 × 256 × 3 tensors and normalized using the mean and standard deviation of the ImageNet dataset. The model was trained for up to 30 epochs with a batch size of 16 images, using the Adam optimizer with a learning rate of 1e-4. Early stopping was applied by monitoring validation loss, and training was halted if there was no improvement after 5 epochs. Training was conducted on an NVIDIA Quadro RTX 6000 graphics card (NVIDIA, Santa Clara, CA, USA) with CUDA Toolkit 11.8 using the Pytorch framework [28], on a workstation with AMD processor and 128 GB RAM. This allows to process a single image within 5–15 ms.

### Evaluation and explainability

The classifier's performance was monitored on the validation set with early stopping applied after 5 epochs of no improvement. The resulting model was then evaluated on the test set using metrics such as accuracy, precision, F1-score, sensitivity, and specificity. Precision, the proportion of correct positive identifications, and sensitivity (or recall), the proportion of actual positives correctly classified, were calculated. Additionally, the confusion matrix, Precision-Recall Curves, and Receiver Operating Characteristic (ROC) curves were computed for each class.

To interpret the model's outcomes, three XAI methods were employed, each providing different visualizations such as heatmaps that highlight areas relevant to the model’s decisions. For a given classification, input images were decomposed into signals (relevant pixels) and distractors (irrelevant pixels). Relevance or attribution, reflecting the importance of each pixel for the classification, was also calculated, as not all pixels contribute equally. Relevance was assessed for either the input image or the signal if available.

Three interpretability algorithms were employed in this study to enhance understanding of the decision-making process. The PatternNet algorithm [16] estimated the signal from the input image, highlighting relevant pixels. The PatternAttribution algorithm provided relevance scores for theses pixels using a color scheme where red indicated high relevance and blue indicated lower relevance. Additionally, the Layerwise Relevance Propagation (LRP) algorithm [8] assessed the relevance of input image pixels without separating the signal from the distractor. These complementary methods were selected to provide a comprehensive understanding of the model’s predictions and to support the primary aim of creating an efficient, interpretable model for training purposes. By combining them with a standard classifier for the classification of sagittal malocclusions from colored intraoral photographs, the approach also highlights key image regions in the decision process—a combination not previously applied to Angle classification.

## Results

**Fig. 4:**
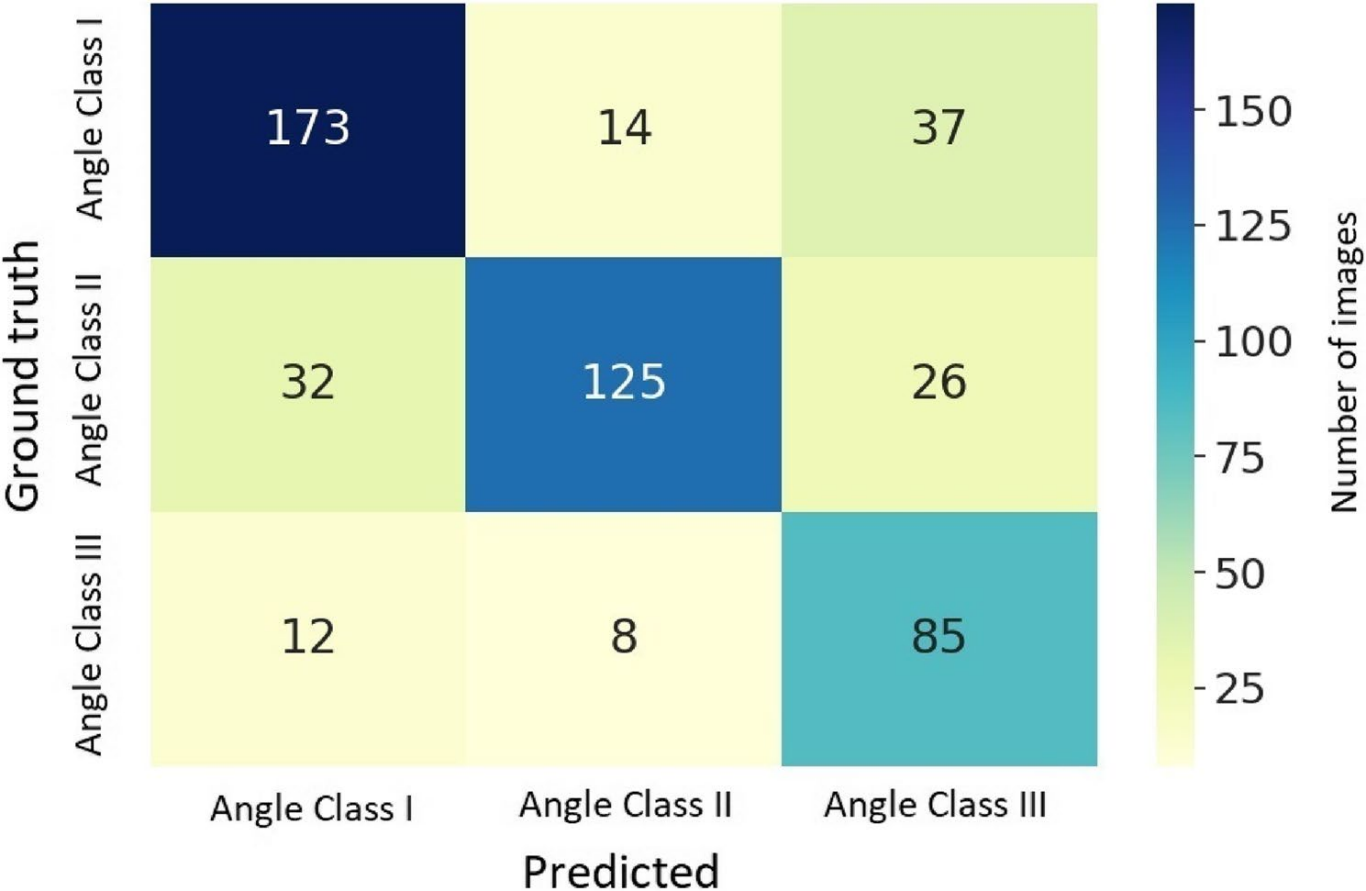
Confusion matrix displaying the classification performance of the model for each Angle class. The main diagonal elements represent the number of correctly classified instances for each Angle class (i.e., the true positives). The off-diagonal elements indicate the misclassified instances, showing how frequently one class was incorrectly predicted as another. This matrix provides a visual summary of the model's accuracy, highlighting both correct and erroneous predictions across the different Angle classes

The model achieved an accuracy of 0.75. Precision and sensitivity were similar at 0.74 and 0.76, respectively. Specificity was 0.88, and the F1-score 0.74. The confusion matrix (Fig. 4) reveals the distribution of correct and misclassified images across Angle classes. Angle Class I images were correctly identified in 77%, while Angle Class II images were more often misclassified (68%) as Angle Class I rather than Angle Class III. Angle Class III images were most accurately classified (81%) and if misclassified, then most frequently as Angle Class I. Overall, misclassification as Angle Class III was most common.

**Fig. 5:**
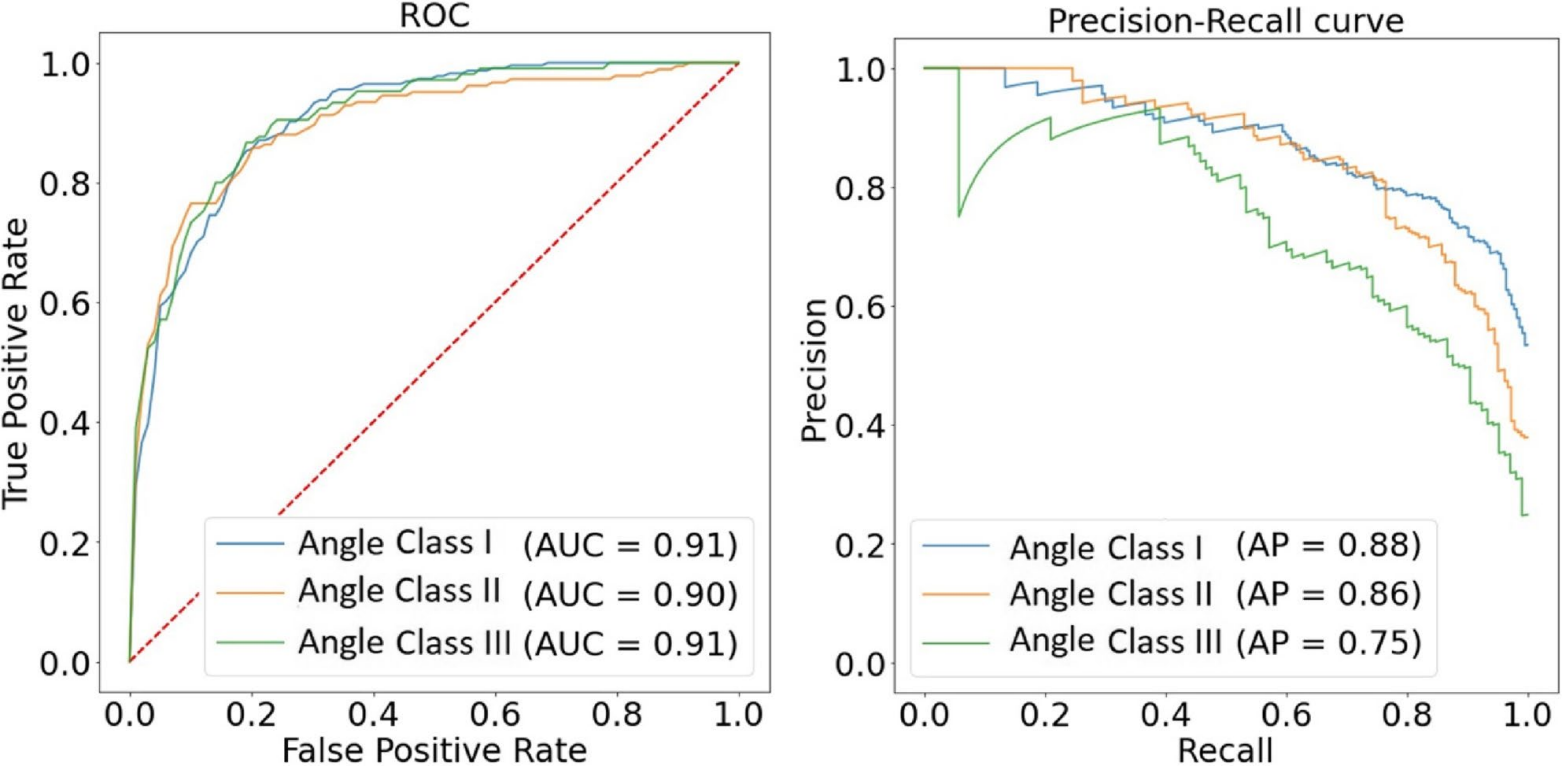
Left: Receiver Operating Characteristic (ROC) curve for each Angle class, illustrating the trade-off between the true positive rate (sensitivity) and the false positive rate (1-specificity) at various threshold settings. The Area Under the Curve (AUC) is displayed, with higher AUC values indicating better classification performance. The dashed red line represents random classification, serving as a baseline for comparison. Right: Precision-Recall curve for each class, showing the relationship between precision and recall at different confidence score thresholds. Precision is plotted on the y-axis, while recall is plotted on the x-axis. The Average Precision (AP) is calculated as the area under the precision-recall curve, providing an overall measure of the model's ability to correctly identify each class while minimizing false positives

Precision-Recall curves (Fig. 5) show that classification performance was highest for Class I, followed by Class II. Class III had the lowest precision due to a high number of false positives.

The Receiver Operating Characteristics (ROC) curve (Fig. 5) provides a visual metric for evaluating classification performance of the models, with the Area Under the Curve (AUC) values for each class included. It shows a strong perfomance of the investigated models.

**Fig. 6:**
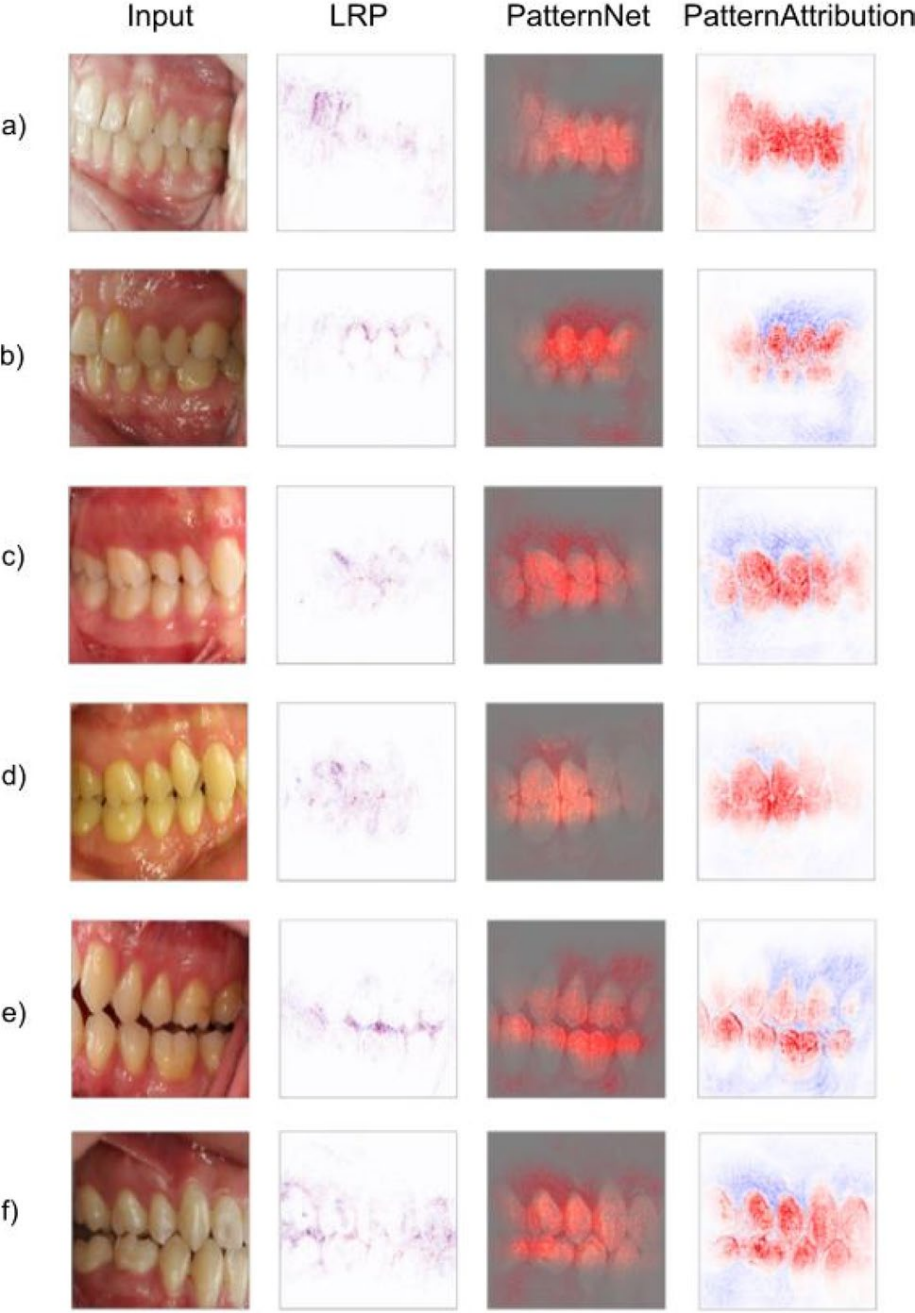
Exemplary images and their interpretability maps by the LRP, PatternNet and PatternAttribution algorithms. **a** and **b** Class I, **c** and **d** Class II and **e** and** f** Class III. LRP outputs a relevance score for each pixel, PatternNet those pixels considered relevant and PatternAttribution a relevance score for the pixels selected by PatternNet

A set of examples of input images and the results from the XAI analyses is presented (Fig. 6). In general, the combination of PatternNet and PatternAttribution proved to be the most informative from a clinical perspective, as it effectively isolated different structures such as teeth and gingiva while highlighting the interdigitation between antagonistic teeth. LRP successfully emphasized the molar area in some cases, although overall explainability was lower. For instance, in the example shown (Fig. 6a), the densest regions of LRP were around the incisors, with only a small highlight in the molar region. In contrast, PatternNet identified the molar area as the primary signal, with relevant pixels concentrated around the molars and extending to the premolars. Similarly, PatternAttribution assigned high relevance scores to the teeth, with greater density observed around the molars.

## Discussion

Automated classification of Angle classes using photographs and AI offers several advantages over traditional clinical examination or model analysis. While Angle classification can be easily determined through these conventional methods, the use of intraoral photographs in combination with AI enhances diagnostic precision and objectivity. The integration of AI reduces inter-examiner variability, ensuring more consistent and accurate assessments, which is especially valuable in orthodontic practices and educational institutes with multiple clinicians of varying experience levels. Furthermore, AI-based classification can standardize the diagnostic process, making it more reproducible across different practitioners and institutions. The use of standardized intraoral photographs also allows for a more detailed and accessible record of the patient’s occlusion, which can be useful for longitudinal monitoring and remote consultations. In clinical training, AI offers the benefit of providing immediate, data-driven feedback to graduate and postgraduate students, as well as young practitioners, thereby accelerating learning and improving clinical decision-making. Additionally, AI-based classification can aid in developing more personalized treatment concepts by automatically analyzing the specific malocclusions present, ultimately contributing to more tailored and effective treatment plans.

Based on the performance metrics obtained, our AI model demonstrated sufficient diagnostic capability in classifying sagittal dental malocclusions according to the Angle classification. However, compared to the study by [7], which reported a classification accuracy of 93.1%, our model achieved a lower accuracy of 75%. Several key differences in methodology, dataset composition, and model architecture may account for this discrepancy, highlighting potential areas for improvement in future research. One notable distinction between the two studies is the classification criterion. While Angle’s original classification is based solely on the sagittal relationship of the first molars, Bardideh et al. [7] additionally included the canine relationship into their assessment. However, this approach does not align with Angle’s definition, as the canines position are subject to variations influenced by the overall Bolton ratio and anterior crowding. Additionally, their classification method included frontal views of the occlusion, which are not traditionally used for Angle classification but may aid in differentiating between Class II and III cases due to the visibility of an extensive positive or negative overjet. In contrast, our model relied strictly on lateral intraoral photographs, focusing on the molar relationship alone. Another important difference lies in dataset size and variability. While Bardideh et al. [7] utilized a smaller dataset than ours, reducing the likelihood of misclassification and increasing model precision, they also employed extensive image augmentation techniques, which likely enhanced model generalization. Furthermore, although both studies were single-center investigations, their dataset included images captured under various lighting conditions and camera settings, potentially increasing model robustness. In contrast, our study maintained stricter standardization in image acquisition but with a broader dataset, which may have introduced additional complexity and classification challenges. Moreover, Bardideh et al. [7] employed a more elaborate AI model using a multiphase approach. Their system first segmented specific teeth from the gingiva before evaluating the occlusal relationship. This additional step provided more localized diagnostic information, which may have contributed to their higher accuracy. In contrast, our model classified the occlusion directly from raw intraoral photographs without prior segmentation, which could explain its comparatively lower performance.

An intriguing aspect of their study is the comparison between AI performance and expert clinicians. Their AI system outperformed the orthodontic specialist in occlusion classification, which raises questions about discrepancies in human assessment. This could be due to variations in the definitions of malocclusions, as expert evaluations are often based on physical model analysis or clinical examination rather than static photographs. Differences in bite positioning at the time of image capture versus impressions and bite registrations could have also influenced the results. This aligns with the known challenge in clinical orthodontics, where transient “Sunday bites” can create inconsistencies between photographic and clinical assessments.

Notably, however, the yielded performance metrics in our study do not fully reflect the risk of shortcut learning, where a model learns to solve a task using incorrect features. Interpretability methods can help identify this and other types of biases, which is why three different XAI algorithms were employed to comprehend which pixels in the photographs were most relevant for determining the Angle class. Though current explainability tools offer only a visual approximation of the network’s decision process rather than a formal, causal explanation, each XAI method highlighted different structures influencing the model's decision-making, offering valuable insights into its functioning. Notably, none of these methods relied solely on the sagittal relationship between the upper and lower first molars, as defined by Angle. Angle’s original definition also included an imaginary reconstruction of the occlusion by the clinician, accounting for the mesial shifting of teeth during the transition from deciduous to permanent dentition. This interpretative task, however, was not required of the algorithms in our study, which were tasked solely with identifying the sagittal occlusion visible in the photographs – a limitation worth noting. However, in addition to the molar relationship, the presented XAI methods also seem to incorporate information from the occlusal relationship of the adjacent premolars (Fig. 6). PatternNet focuses mainly on the posterior teeth, particularly the molars and premolars with strong density around key regions critical for determining the occlusal relationship according to Angle, including the cusps and their interdigitation. PatternAttribution provides a broader segmentation of all visible teeth and surrounding tissues, distinguishing teeth as relevant structures highlighted in red, while less relevant regions, such as gingiva and other soft tissues, are marked in blue (Fig. 6). LRP highlights the separating lines and spaces between various structures, such as interocclusal spaces and gingival margins that delineate the gingiva from adjacent tooth surfaces (Fig. 6). Its focus is not strongly concentrated on specific teeth or regions, resulting in less distinct isolation of occlusal features. A notable limitation observed in all XAI methods employed in this study is that the region of interest – specifically the sagittal molar relationship – was not explicitly defined or narrowed in the photographs. As a result, the XAI models primarily demonstrate an ability to separate different anatomical structures and perform a structure segmentation rather than accurately localizing the precise region responsible for the correct classification, as originally proposed by Angle. Despite this limitation, the model demonstrates sufficient accuracy in determining the Angle class. The specific factors contributing to this success, however, remain unclear and involve the intercuspation of additional teeth beyond those traditionally considered in Angle's classification. Interestingly, the misclassifications observed offer valuable insights into both clinical and technical reasons underlying these errors, which could guide future improvements in model design and diagnostic accuracy.

**Fig. 7:**
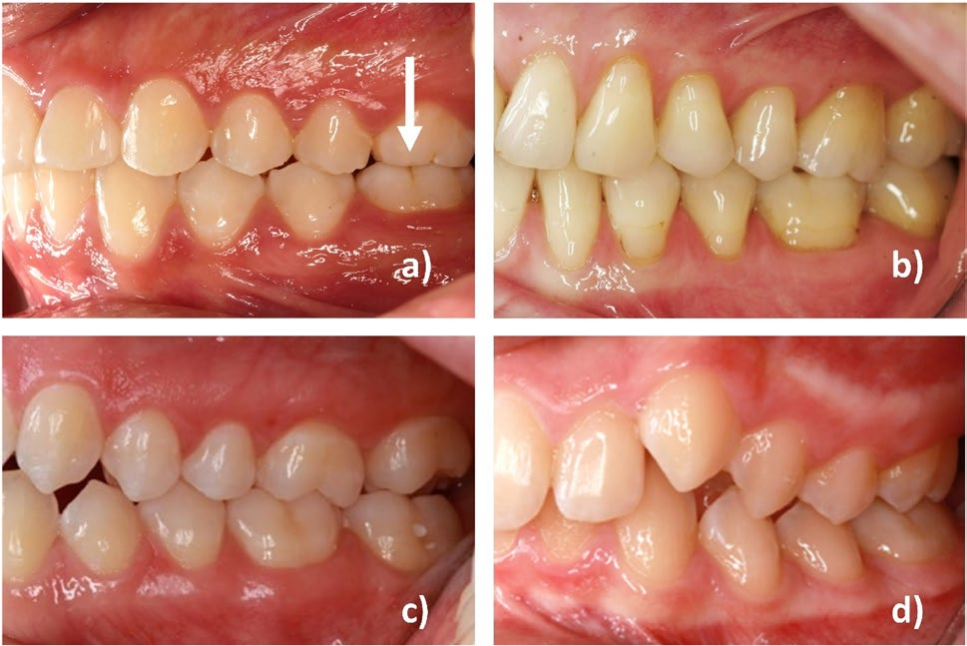
**a** Displayed Angle class I occlusion with minor tooth abrasions of the cusps of the first upper molars contributing to misinterpretation of the AI-based algorithm as Angle class II occlusion; **b** Misclassification of an Angle class III as Angle class I due to a divergent Bolton Ratio; **c** An almost 90° angle of the camera lens to the sagittal occlusion as example of an useful intraoral photograph; **d** Example of a misclassification of an Angle class II as an Angle class I due to a too acute angle of the camera lens to the sagittal occlusion

Interestingly, the misclassifications observed provide further insights into potential clinical and technical factors contributing to the errors. Clinically, misclassifications were more likely in cases where the malocclusion was minimal, either in the mesial or distal direction, or when the mesiobuccal cusp of the upper first molar deviated only slightly from the buccal pit of the lower first molar due to angulation. Contributing to the large share of misclassifications were also teeth with minimal signs of tooth wear of the relevant cusps. The latter is relevant because dentitions that were severely affected by tooth wear were originally excluded from the study. Even minor tooth wear appeared to challenge the model, as it struggled to accurately identify the cusp tip and subsequently interpret its position and intercuspidation to the antagonist tooth (Fig. 7a).

Additionally, our XAI methods revealed that dentitions with severe anterior crowding or Bolton ratio discrepancies were prone to misclassification. These issues typically stem from differences in the mesiodistal dimensions of the maxillary and mandibular teeth. As a result, dentitions with divergent tooth sizes between corresponding antagonists often exhibited malocclusions, which became more pronounced in the distal regions when no spaces were present in one dental arch. This frequently led to cases where a proper sagittal relationship was observed at the canines, while malocclusion began with the premolars and was most pronounced at the molars (e.g., Angle Class III, Fig. 7b).

A common technical reason contributing to the misinterpretation of Angle classes was the varying angulation of intraoral photographs taken by clinicians using a mirror. Despite prior instruction, standardized parameters for taking photographs with the specific camera in the department, and practice sessions, variations occurred due to either the patient's limited mouth opening, individual soft tissue constraints, or the clinician’s learning curve in photography techniques during a clinical setting. To avoid causing discomfort to the patient in such cases, clinicians likely captured the photographs at an increasingly acute angle towards the sagittal occlusion from the front. This may have resulted in more frequent misclassifications in the respective photographs, particularly when misinterpreting Angle Class II classifications as Angle Class I (Fig. 7d). This coincides with previous data [15] showing that only 80% of participating clinicians correctly judged the patient’s occlusal relationship when photographs were taken from an inadequate angle, highlighting the increased difficulty in challenging intraoral situations and the importance of experience in taking intraoral photographs. High-quality intraoral photographs are essential for an accurate diagnosis.

This study contributes to the exploration of explainability methods for AI-based analysis of intraoral photographs by employing three distinct XAI methods and conducting a detailed evaluation of model misclassifications. The insights gained from these analyses advance the understanding of how AI models function in orthodontic diagnosis and highlight areas for improvement. Class imbalances in the dataset for model development were addressed through use of a weighted cross-entropy loss function. However, our study also presents certain limitations. The dataset for this study was collected at a single clinic and involved different clinicians with varying levels of experience in taking intraoral photographs, despite having received initial instruction and practice sessions. We further cannot claim generalizability, particularly with respect to different populations, varying camera types, and photo qualities. While the study's single-center design allowed for strict standardization of image acquisition and classification, minimizing variability, future research should explore multi-center datasets to enhance the generalizability of AI-based Angle classification.

In this study, we did not conduct an extensive hyperparameter search – a complex and computationally intensive process of fine-tuning model parameters. This decision was justified, as our primary focus was not on optimizing the clinical performance of the model but rather on exploring its general properties and the inherent logic underlying its classification decisions. However, in a separate investigation, we optimized hyperparameters for deep learning-based classification of orthodontic photographs along the Angle classes, achieving a model with sufficient accuracy at a learning rate of 1–3 × 10⁻⁶ and a batch size of eight, with automatic image augmentation improving all metrics by 5–10% [10]. Given the focus of the current study was on applicability and explainability of the model rather than hyperparameter tuning, we did not perform cross-validation, commonly used to ensure a model’s generalizability across different data subsets. Instead, we prioritized the direct applicability of XAI methods, as the performance of the initial algorithms was deemed sufficient for the purposes of this study. To further reduce misclassification errors, future studies should explore data augmentation strategies, including synthetic image generation and occlusion handling. Additionally, refining the model architecture may enhance robustness in challenging intraoral conditions.

Also, we must acknowledge potential limitations related to the generalizability of our findings due to our single-center dataset. Models trained and validated solely on single-center data might perform less reliably when applied to external datasets or different institutions, underscoring the need for multi-center validation to ensure robustness and wider applicability.

## Conclusion

The developed AI model sufficiently classified sagittal dental malocclusions into Angle Classes I, II, and III using common intraoral photographs. Three XAI methods were introduced and displayed the model’s decision-making: LRP focused on interocclusal spaces and interdigitation, PatternNet emphasized the posterior dental region, and PatternNet Attribution distinguished teeth from surrounding soft tissue.

AI can support clinicians, researchers, and educators in classifying Angle classes, while XAI methods help scrutinize the model’s logic. Using multiple XAI approaches may enhance explainability. Future work should address clinical and technical limitations, enhance model interpretability, and ensure greater generalizability across diverse datasets and imaging conditions.

## Data Availability

The data presented in this study are available on request from the corresponding author. The data are not publicly available due to data protection reasons.
